# Ways into physical (in)activity: The role of critical life events and transitions in the reconstructions of young adults

**DOI:** 10.1371/journal.pone.0290438

**Published:** 2023-08-22

**Authors:** Hannes Gropper, Jannika M. John, Ansgar Thiel

**Affiliations:** 1 Institute of Sports Science, Eberhard Karls University, Tübingen, Germany; 2 Interfaculty Research Institute for Sport and Physical Activity, Eberhard Karls University, Tübingen, Germany; University of Illinois at Urbana-Champaign, UNITED STATES

## Abstract

**Background:**

Physical activity (PA), sport, and exercise levels generally tend to fluctuate or decline from childhood to adulthood. Life events and transitions may have a positive or negative impact on these behaviors. However, most research in this regard is quantitatively framed and we know little about why and how life events and transitions affect PA-related behaviors.

**Objectives:**

The aim of this study is to understand when, why, and how individuals change their daily PA, sport, and exercise behaviors and related attitudes in the context of life events and transitions and what underlying dynamics promote these changes from a subjective perspective.

**Methods:**

Forty-six young inactive adults (33 women) aged between 20 and 40 years from the iReAct study participated in a mixed-method biographical mapping, which combines a semi-structured interview with a drawing activity to explore subjective experiences of life events, transitions, and PA-related behaviors across the life span. To analyze data, we conducted a reflexive thematic analysis.

**Results:**

We developed three overarching themes that reflect the underlying dynamics which are associated with the occurrence of critical life events and transitions and lead to subsequent changes in daily PA and sport and exercise behavior: (1) *The finitude of temporal resources*; (2) *The plasticity of priorities and motives*; and (3) *The (in)conveniences of context*.

**Conclusion:**

Our results illustrate that there is not a straight causal link between life events and behavior. Rather, critical life events and transitions can have a tremendous impact on temporal resources, individual priorities and motives, and contextual factors, such as the physical and infrastructural environment and social settings and relationships. Consequently, more or less extensive changes in those circumstances can lead to individual adaptations in daily PA or sport and exercise and related attitudes, as they require individuals to re-balance behavioral patterns.

## Introduction

While physical activity (PA), exercise, and sport play central roles in some people’s lives, many more hardly spend any time being physically active. A broad body of research has shown that oftentimes neither adolescents nor adults meet recommended minimum PA levels [1, 2] and that inactivity generally tends to rise as individuals age [3]. Considering the potential developmental and biopsychosocial benefits of regular PA, sport, and exercise [4–7], it is one of the central goals of researchers, health professionals, and policy makers to understand how individuals come to live an active or rather an inactive life.

Recently, several literature reviews have shown that life events and transitions across the life course may have an impact on PA, sport, and exercise behavior in both positive and negative ways [8–10]. Although findings are partly inconclusive [10], the period of becoming an adult, in particular, has been identified as a time during which life events and transitions (e.g., starting cohabitation, getting married, starting to study, entry into the labor market, pregnancy, and parenthood) are mainly associated with declines in PA levels. Moreover, educational transitions during childhood and adolescence (e.g., from kindergarten to primary school to secondary school) have similarly been pointed out as critical periods, during which PA is likely to decrease [9–15]. Yet, besides the realization that “how individuals adapt to the same event is a highly idiosyncratic process” [10], most of these reviews could not provide any *actual* explanations for those trends.

Theoretically, researchers have considered that life events and transitions might lead to environmental and institutional changes [16–19], reallocations of available resources, and shifts in personal priorities [20–22], which, in turn, alter PA, sport, and exercise behavior. Likewise, the co-occurrence and accumulation of various life events may shake up daily routines leaving individuals more vulnerable to abandon behavioral patterns [9, 22]. On the other hand, PA-related activities might act as coping mechanisms and thus be taken up or increased in response to stressful life events and transitions [23, 24]. Yet, despite those deliberations, empirical research on *why* and *how* life events and transitions affect PA, sport, and exercise behavior remains scarce. Most studies investigating trajectories of these activities across the life span employ prospective longitudinal and mostly quantitative designs [see also 10] that allow to identify temporal trends on a more global level, but cannot provide a deeper understanding of the underlying dynamics of those tendencies. Thus, little is known about the different ways in which individuals adapt their behavioral patterns as a response to similar life events and which PA domains (e.g., daily PA, sport, or exercise) are actually affected by life events [10, 23].

Only few studies employ qualitative approaches and help understand why and how life events and transitions affect PA-related behavior. Yet, these studies often focus on pre-selected life events or transitions [e.g., 25–27] or assess middle and older aged samples [e.g., 28, 29]. We are aware of only one qualitative study that examined *various* life events and their impact on sport behavior in young adults [30]. On the one hand, these studies yielded some interesting insight into which dynamics *actually* lead to behavioral adaptations, such as changes in social networks or living surroundings [29] or in temporal, social, physical, mental, and economic resources and individual priorities [30]. On the other hand, studies exploring which life events and transitions people *subjectively* experience as significant with regard to their PA, sport, *and* exercise behavior and why and how these events and transitions altered their behavioral patterns are scarce.

In light of the current state of research, we aim to address three desiderates in this article. First, by employing a qualitative approach, we aim to understand which life events and transitions young adults themselves consider to be *subjectively relevant* for their PA behavior over the life span instead of using pre-defined life event scales. Second, in order to provide possible explanations for behavioral changes, we are interested in unraveling the *underlying dynamics* which promote these changes. In this regard, we focus on the questions of when, why, and how different PA-related behaviors, such as daily PA, exercise, and sport, change as life events and transitions occur. Herewith, biographical approaches offer a highly valuable perspective, as they allow for a retrospective evaluation of past experiences and events. Third, a focus on the group of inactive *young* adults is particularly interesting, as previous research has shown that life events and transitions from childhood to young adulthood are often strongly associated with declines or at least fluctuations in PA, sport, and exercise behavior [3, 31–33]. Thus, with our study, we aim to explore how young inactive adults retrospectively make sense of life events and transitions in relation to their PA, sport, and exercise behavior. In this regard, we are especially interested in how they explain behavioral and attitudinal changes in order to gain a better understanding of the underlying or associated dynamics leading up to those adaptations.

## Theoretical considerations

Research on life events has commonly been framed from either a stress perspective [34] or a developmental perspective [35, 36]. Characteristically, a stress-oriented approach conceptualizes life events as external negative stressors that are attributed a pathogenic effect in the etiology of diseases and disorders, in particular, as they accumulate over time [37]. From a developmental perspective, life events are, on the other hand, not necessarily pathogenic in nature. Rather, they can be considered as natural interventions that occur across the life span, which may account, in a broader understanding of the terminology, for development and change over time [10, 37]. This is not to say that similar life events lead to similar adaptions across individuals. Rather, the individual response to life events depends on factors like their timing in the life course, whether they are normative or non-normative and more or less foreseeable, sudden, or severe. Therefore, it is crucial whether and to which degree a life event is valued as personally significant–meaning whether it is considered to be *critical* [37]. Eventually, *critical* life events can be conceptualized as life changes which influence daily behavior and demand adaption in order to reestablish a person-environment fit or to (re-)adjust behavioral patterns, such as PA, exercise, or sport [35, 38].

While we conceptualize critical life events as singular instances that may “mark the beginning or the end of a specific status” [39, p. 4], they do not occur in a socio-historical vacuum. Rather, critical life events are embedded in the life course and have a biographical history and aftermath [10, 36]. The previously mentioned terms adaption and (re-)adjustment indicate that critical life events may trigger processes that have to be considered in their temporal dimension. To account for the temporal character of the adaptive process and adjustment periods related to life events, we refer to the concept of *transitions*. Transitions temporally exceed the duration of a critical life event, yet both are “two sides of the same experience” [10, p. 3]. In this regard, critical life events may either *trigger* transitions or occur already *within* the transitioning process, as a cognitive pre-occupation might already begin before a critical event takes place–depending on whether an event can be more or less anticipated. The temporal structure of a transition, in turn, is dependent on the degree of *social readjustment* [40] that is necessary to adapt to a new status as new networks, new behaviors, and new self-perceptions and roles emerge [41]. Critical life events and transitions may thus alter behavioral patterns, such as PA, sport, and exercise, especially since these behaviors have been associated with a multitude of biopsychosocial correlates and determinants [42–44] that might change in importance across the life span [45]. Similar to critical life events and transitions, PA, sport, and exercise are subjective phenomena that are inherently cerebral, social, situated, and political [46]. Moreover, these phenomena are highly complex and multidimensional as they can occur at different intensities and across different settings [47–49]. For this paper, we focus especially on two behavioral patterns relating to PA. First, we will look at daily PA, which includes activities such as active commuting (e.g., walking or cycling), occupational PA, domestic activities, and leisure activities at light intensities (e.g., walking, playing). Our second focus will be on exercise and sport as activities that that are planned, organized, structured, and repetitive with the aim to increase or maintain physical fitness or performance or to participate in competitions [47, 48]. It is important to note that in Germany, where this study was conducted, the term *sport* has a more universal meaning than in English-speaking countries and conflates with the term exercise (which has no equivalent translation in German). For ease of presentation and readability, we will mainly refer to the terms sport or sport activities and subsume exercise under this term if not otherwise stated.

On a methodological level, our theoretical considerations on the experiential subjectivity of critical life events and transitions and daily PA and sport and exercise behavior imply the necessity of qualitative approaches in order “to provide rich description and possibly explanations of people’s meaning-making–how they make sense of the world and how they experience particular events” [50, p. 10]. In this regard, biographical approaches offer an exploratory avenue that allows assessing individual and subjective experiences of critical life events and transitions in relation to daily PA and sport and exercise behavior and the interpretation of those experiences [37, 51]. Thus, with this study we aim to answer the following research question: What are the underlying dynamics associated with critical life events and transitions leading to changes in daily PA and sport and exercise behavior?

## Material and methods

This research project was one of five subprojects in the larger interdisciplinary *Individual Response to Physical Activity (iReAct)* study [52]. At the baseline measure of this 15-week two-period sequential intervention study, among others, biographical mapping interviews were conducted to explore how physically inactive young adults subjectively reconstruct PA- and health-related experiences and which meaning they attribute to life events and transitions in this regard. In line with our theoretical considerations on the subjective nature of critical life events, transitions, and PA and exercise, the research of this subproject falls within an interpretivist paradigm and is underpinned by ontological relativism, assuming that reality is multiple, individually constructed, and, thus, mind-dependent [see also 53, 54]. Additionally, we assume that “any narrative account of personal memory is created within a specific situation, by particular individuals, for particular audiences, and to fulfill particular goals” [51, p. 262]. In this regard, the narrative accounts of biographical experiences must be considered a co-construction of the interviewer and the interviewee that is situated within a particular context (i.e., the iReAct study) and centered on a particular topic (i.e., biographical experiences of critical life events and transitions and PA and sport behavior).

### Sampling and procedure

After approval by the “Ethics Committee of the Medical Faculty University Tübingen” (reference number: 882/2017BO1) inactive, yet healthy, young participants were recruited using several channels, such as the university mailing list, the university clinics mailing list, postings on an experimental database, newspaper articles, online presence on the homepage of the institute of sports science, and flyers. The detailed inclusion and exclusion criteria and the invitation process for the participation in the iReAct study have already been described elsewhere [52]. For this paper, the final sample consisted of 46 inactive participants who were aged between 20 and 40 years. This age category was chosen as it relates to the periods of emerging and young adulthood [55], which were relevant for the iReAct study. Thirty-three of the participants were women. All participants gave informed consent to participate in all study modules and to audiotape their interviews.

For the interviews, we used the biographical mapping method, which is a mixed-method approach that combines a semi-structured interview with a drawing activity and that allows to explore people’s subjective experiences of PA, exercise, and health across the life span [for a detailed description, please see 56, 57]. During the interview, participants are asked to fill in a two-dimensional grid together with the interviewer. The horizontal grid line is customized and represents the respective participant’s life span in years. The vertical line represents an intensity scale ranging from zero to ten and allows participants to draw behavior- and state-related trajectories and rate those experiences at different times of their life.

During the first part of the interview, participants were asked to reminisce about relevant life events and transitions that they could remember and that made them who they are today. This was purposefully asked in an open manner to include all the *critical* life events that were important to the participants and not just those related to PA or health. Critical life events and transitions were written down on the x-axis of the two-dimensional mapping grid by the interviewer, while participants elaborated on the relevance of the life events for their development. At the end of this part of the interview, the interviewer asked about more specific events, relating to PA, exercise, and health, but also to leisure-time activities, social context, or education and employment.

In a second step, participants were asked to plot 15 different developmental lines (for an overview of all assessed dimensions, please see S1 Table), which centered, among others, around the subjective amount of daily PA and the subjective amount, relevance, enjoyment, and temporal resources for exercise and sport behavior across the life span. Moreover, the interviewer invited the participants to orient themselves towards the critical life events and transitions noted on the x-axis and to think-aloud and comment on the trajectories while drawing. For each dimension, a new *blanco* grid was used, which only displayed the critical life events and transitions but not the previous curves. When participants remembered further events and transitions that were not previously mentioned, those events were added to the timeline and participants had the chance to go back to previous curves to adjust the trajectories if necessary. During the interview, the interviewer asked questions when uncertain why turning points occurred or when participants became too immersed in the drawing activity and stopped talking. After the interviews, the interviewer took field notes and filed a short protocol sheet. The biographical mapping interviews lasted between 55 and 116 minutes with an average length of 83 minutes. HG conducted all interviews.

### Analysis

For our analysis, we focused primarily on the interview data from the mapping. All interviews were transcribed verbatim by HG and trained research assistants, anonymized, and imported into MAXQDA [58]. To explore the patterns of shared underlying dynamics associated with critical life events and transitions leading to changes in daily PA and sport and exercise behavior as well as related attitudes, HG carried out a reflexive thematic analysis that focused on participants’ subjective experiences [59–62]. In line with our ontological position, the data analysis was considered to be a “meaning-making process” [63, p. 5] acknowledging that our own subjective perspectives diffused into the interpretations and decisions on how to analyze the data [59, 64]. HG is a sport scientist in his early thirties. Throughout his life, and especially during childhood and adolescence, he pursued an active lifestyle and participated and competed in various sports (e.g., handball, basketball, and road cycling). After starting to study, there were more lapses and relapses in his daily PA and sport and exercise behavior that oftentimes occurred in the wake of certain life events and associated life changes (e.g., starting to study and having to commute to university, accidents and injuries, becoming a parent). In his previous research, he has already focused on the impact of life events and transitions on PA and exercise and on subjective experiences of PA and exercise behavior.

In our analytic procedure we oriented ourselves towards the six phases of doing reflexive thematic analysis proposed by Braun and Clarke [62]. Yet, those steps partially intermingled and overlapped as we went back and forth and the analysis grew organically and in a recursive fashion [64]. First, HG familiarized himself with the interviews by reading and re-reading the transcripts and writing down memos on instances that were striking, interesting, or contradictory with regard to the research questions. This included for example instances, when participants described trajectories of PA behavior, when they referred to motivational or attitudinal changes due to life events and transitions, when similar explanations were used across interviews, or when it was unclear what they were trying to say. In a second step, HG started coding the data set in MAXQDA. This was done primarily inductively and HG aimed to code on both a semantic and a latent level. However, due to his existing knowledge (e.g., through conducting a literature review on the impact of life events on PA [10] and our theoretical pre-considerations), coding was also inevitably deductive in some ways. While coding, HG kept writing down considerations and doubts that occurred along the way and taking notes and memos for data items as well as for codes. In addition, he regularly consulted with AT and JJ and other critical friends in order to debate codes and possible interpretations and increase reflexivity [65].

After the first round of coding, HG went through the transcripts for a second time and re-checked and re-coded the data set to counteract what Braun and Clarke [64] have labelled as *coding drift*. In this step, some codes that were semantically the same were already conflated and assorted. In a third step, he printed out all codes and started to explore potential connections between codes and cluster them accordingly. This, similar to coding, was organic and recursive as the codes were laid out on the floor and HG constantly tried to reflect on possible assortments and consulted with AT and JJ to discuss potential candidate themes. During this step, it became apparent that a theme development that centered around single critical life events and transitions was not feasible, primarily because we considered this to reflect merely topic summaries [61, 62]. Rather, we interpretatively started to develop candidate themes that captured the underlying dynamics and mechanisms that seemed to be associated with critical life events and transitions and thus altered daily PA and sport and exercise behavior and related attitudes. These candidate themes were inherently about contradictions as they reflected the ambivalent dynamics that go hand in hand with the occurrence of critical life events and transitions in relation to daily PA and sport and exercise, meaning that similar life events could account for both advantageous and disadvantageous effects among different participants. After having developed initial themes (i.e., bundles of codes), we created a thematic map to get a better overview of our thematic structure and possible associations between the candidate themes. In a fourth step, we reviewed the candidate themes and referred them back to our coding and the data set to refine them if necessary and ensure the coherence of our analysis. Eventually, in the fifth phase, HG wrote up theme names and definitions, carefully trying to make sure that each theme had a central organizing concept and was internally consistent [59, 64, 66]. The sixth, step was drafting the final report. All six phases were not detached from each other, but rather marked by fluidity and by going back and forth in the data set, the codes, as well as the initial and final themes.

## Results and discussion

**Fig 1:**
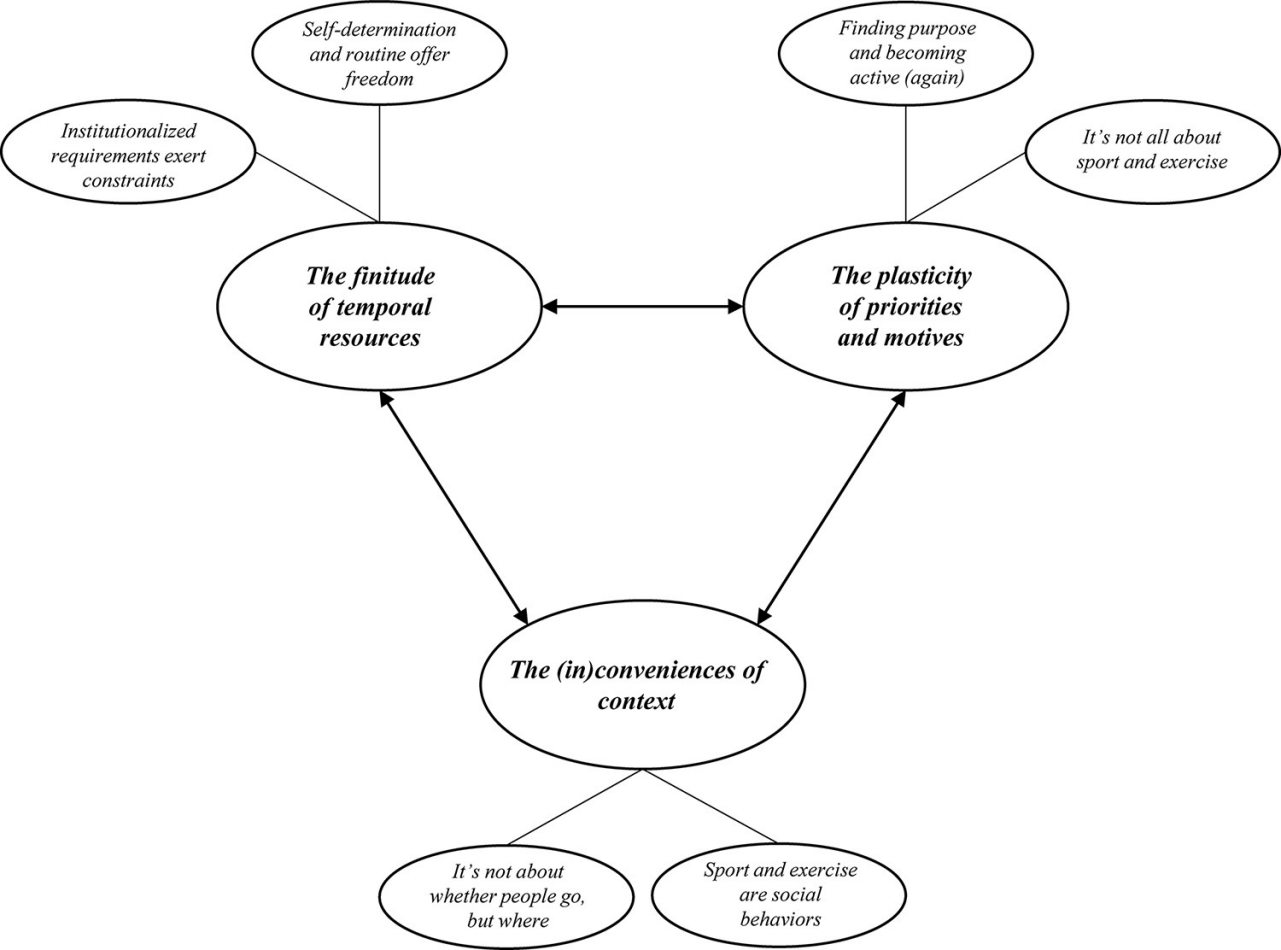
Thematic map.

We developed three themes that reflect our attempt to identify the underlying dynamics that are associated with the occurrence of critical life events and transitions and lead to subsequent changes in daily PA and sport and exercise behavior: (1) *The finitude of temporal resources*; (2) *The plasticity of priorities and motives*; and (3) *The (in)conveniences of context*. Importantly, these dynamics appeared to be inherently ambivalent. While some participants experienced one dynamic as a barrier to daily PA or sport and exercise, others experienced the same dynamic as a promoter. Further, our data indicated that a life event or transition might lead to different dynamics, which, in addition, might be inter-related to each other (see Fig 1).

### Theme 1: The finitude of temporal resources

This theme displays how critical life events and transitions lead to shifts in temporal arrangements and consequently to changes in daily PA and sport and exercise behavior. In this regard, participants indicated, on the one hand, how time is a finite resource and how transitions specifically into highly institutionalized and standardized contexts oftentimes forced them to reallocate this scarce resource away from PA, sport, and exercise, which is displayed in the subtheme *Institutionalized requirements exert constraints*. On the other hand, life events and transitions might also lead to more time, especially when participants transitioned out of an institutionalized context or when institutions either provided clear structures or left more room for self-determination and autonomy. This is captured by the subtheme *Self-determination and routine offer freedom*.

#### Institutionalized requirements exert constraints

Participants generally experienced declines in their temporal resources and subsequently in their daily PA as they transitioned into highly institutionalized and standardized contexts. In this regard, they particularly reflected on educational transitions, such as from kindergarten to primary school, from primary to secondary school, and from secondary school to university, but also into the labor market as incidences when they became less active in daily life [see also 11, 14]. Participants associated these transitions into new institutional contexts with new demands, such as extended school, university, or working hours, more homework or learning obligations, and increased commuting times, which consequently took a toll on their daily PA levels while simultaneously increasing their sedentary time. Sebastian, for example, described his transition to university:

“And then, with the beginning of the study it [daily PA] has decreased further and further (.). Um, because I then simply lacked the time at some point and I was basically more busy with studying (grins)”. (Sebastian)

It is noteworthy, however, that the impact these transitions had on temporal resources depended on the properties and requirements of an institutional context and the associated degrees of freedom. In this regard, participants described for example that daily PA tended to decrease across the transition from kindergarten to primary school, but that there was still time to be outdoors, meet with friends, and play as they used to do in kindergarten. This is in line with previous quantitative research, which found that decreases in PA across this transition occur especially during school, but not during children’s after-school leisure time [17] or the weekend [16] and that overall PA levels are still relatively high at this age [19]. In contrast, Jack described the transition from primary to secondary school and the associated declines in free time and daily PA in the following way:

“Then, yes, I think, at high school, that was probably a slump. Then it goes, um, first down, because just more school, also with school in the afternoon and so”. (Jack)

This in line with research that states that total PA tends to decline across this transition, which is additionally often accompanied by increases in post-school sedentary behavior and screen time [67–69].

The educational transitions from kindergarten to primary school and from primary to secondary school did not only affect daily PA, but also sport behavior as participants experienced significant reductions in their available time for sport activities due to the new demands and obligations. Anne illustrated her transition to a higher secondary school in the following way:

“Then here, at high school, I felt I had less time, because somehow, because for me personally, high school was more difficult than primary school. And then, I just had to learn more”. (Anne)

However, it is important to note that only few participants actually dropped out from their sport activities across these transitions. In this regard, the results may rather have to be considered under the premise that participants experienced less available time, which does not mean they had no time at all. Participants further experienced similar dynamics as they transitioned to university and into the labor market (especially when starting a desk job). Some even indicated a reinforcement of sedentary time, leaving less temporal resources for daily PA, sport, and exercise. In addition, if participants had to spend a full day at work, they were less willing to participate in sport activities since the circumstances might not suit them anymore, such as for example the time of the day, the weather, or the schedule of sport offers. Lisa, for example, described how she regularly came home late from school after starting her apprenticeship:

“And with the training just in the school blocks, I just didn’t have the time. Because then I would have had to drive there in the evening and in winter it was too dark and they didn’t have a riding hall. So it had, it was just impractical from the circumstances. Because I couldn’t have ridden through the forest in the dark”. (Lisa)

In this regard, it seems that the reallocation of temporal resources due to institutionalized requirements takes place first; in a subsequent step, individual priorities, motives, and also possibilities might also become re-evaluated. Hence, while there might actually be some time left, these timeslots of the day might not be the ones when sport activities are most enjoyable or convenient. These findings are in line with previous research that suggests that time-consuming activities, such as becoming a student or starting to work, can lead to a lack of time and individual flexibility, fatigue from work, logistic issues, and reduced opportunities and motivations to be physically active [30, 70–72]. However, we should also point out that, while starting to work often led to less available time for daily PA and sport activities outside of work, some participants also experienced an increase in their daily PA, mainly as occupational PA increased. Those experiences were particularly linked to physically demanding jobs such as starting an apprenticeship (e.g., to become a nurse or carpenter), beginning a civil service (e.g., in caring facilities, hospitals, or patient transport), or side jobs where they had to stand or walk a lot (e.g., sales, postal service, or cleaning).

Similar to starting a full-time job, participants experienced starting a side job as a time consuming endeavor limiting the temporal resources for sport activities. Most participants who described this tendency were enrolled at university while needing to work to finance their studies [see also 30]. Emma, who found herself confronted with multiple tasks and obligations, explained how additionally pursuing a sport activity was impossible for her:

“And then it went downhill, because then, at the beginning of [year], where I had the internship, a part-time job and my studies, I had no time at all. So, there, sport would have been just an additional burden. Even if you only do something for 10 minutes a day somehow. But that was just too much for me, because at times I left the house at 7 o’clock in the morning and came home at half past one. at night”. (Emma)

This is in line with previous considerations that different life domains might compete for temporal resources as life events conflate and thus lead to a termination of leisure-time PA [22].

#### Self-determination and routine offer freedom

While the transitions into institutionalized contexts might mostly limit temporal resources to pursue PA, sport, and exercise, transitioning out of these settings was, in contrast, associated with a substantial increase in available time as requirements and duties dispersed. Participants described this feeling of new freedoms especially in the context of events, such as graduation or going abroad but also towards the end of their study. Those events share the commonality of decreased obligations and are associated with a sense of freedom and available time for oneself. However, these occasions are often short-term periods that are usually followed up by transitions into a new context (e.g., university or work) and the adoption of new roles, which are likely to decrease the available time again. Additionally, having more time does not necessarily mean that people actually pursue sport or exercise activities, especially if it is not important to them (e.g., when going abroad). We will elaborate further on this last point in the theme *The plasticity of priorities and motives*.

Some participants experienced an increase in the available time for sport activities as they started to work as it provided them with a clear daily structure and an end to the working day, as can be seen in George’s statement:

“I have to say that I have a bit more free time now while I’m working now, than during my studies. (..) Towards the end, after my exams, I had more time. I had fixed working hours”. (George)

In addition, flexible working hours and more freedom were perceived to allow more time for sport activities. In those instances, it might be more appropriate to speak of institutionalized opportunities than of constraints. Ben described his workday and the associated freedom during his civil service in the following words:

“And then the civil service, that was a dream. It was just great. So I had, I think around at half past three closing time and was then just free from everyday life’s burdens”. (Ben)

Similarly, several participants experienced their transition to university as a period when temporal resources became available again, as Michael, for example, indicated:

“Then came my undergraduate studies. Yes, I think probably already more time, I mean humanities are not so lecture-intensive at first, I must admit (grins). Um that is, yes so that is the normal ranking, I would have had time, in any case”. (Michael)

This is in line with previous findings, which suggest that starting university may be experienced as leading to an increase in personal autonomy and, thus, in temporal resources and flexibility [30].

Finally, while transitions into institutionalized contexts generally impair temporal resources, these limitations do not necessarily have to persist over time. For example, while participants often experienced a decline in available time across their transition to secondary school, some of them also experienced an increase again, indicating that they had developed a routine and became able to re-balance their temporal resources and make more time for daily PA and sport activities. Anne, for instance, indicated:

“And then at the new school, I don’t know, you got used to it. I didn’t learn that much anymore. And (…) I rode my bike again”. (Anne)

So-called rebound effects have similarly been described for the transition to primary school [18, 73] or the post-partum period [74, 75]. This is important insofar, as it shows that some behavioral changes, such as declines in daily PA and sport behavior might occur only as short-term adaptations until new routines have been established and as a result free up temporal resources.

### Theme 2: The plasticity of priorities and motives

This theme summarizes how the experience of critical life events and transitions leads to fluctuating priorities and motives especially with regard to sport and exercise behavior. We identified two opposing dynamics. First, critical life events and transitions can shift priorities away from sport and exercise as other life domains become more important, which we elaborate on in the subtheme *It’s not all about sport and exercise*. Second, finding a purpose in sport and exercise due to the occurrence of critical life events and transitions can increase the relevance of these activities as they become a new or reinstated priority, which we discuss in the subtheme *Finding purpose and becoming active (again)*.

#### It’s not all about sport and exercise

As participants experienced new priorities or interests in other life domains, sport activities oftentimes lost relevance for them. Most prominently, participants deemed graduation from secondary school or university as well as examination periods and final theses to be more important than sport and exercise and were thus willing to invest more time into their studies. Here, in contrast to the previous theme, they re-allocated temporal resources due to a change in priorities and not the other way around. As participants consequently experienced a time tradeoff between learning on the one hand and sport activities on the other, they often decreased or dropped out from the latter. Mandy, for instance, explicated how sport became less important to her as she approached graduation:

“At some point it [sport] was important to me … And then, towards graduation, it wasn’t important at all, because I just wanted a good grade”. (Mandy)

When something more important came up, this tendency might be even more pronounced as some participants argued. Often, at these times, their enjoyment of sport and exercise decreased as these activities generally require time commitment and some level of organization, which participants perceived as an additional stressor [see also 72]. Emma, for example, stated:

“Exactly, I also did that [sport] with a friend and we also always went there together. But then that just, we stopped, yes both of us, because then the exam period had started and that was too stressful for us and that was then just the easiest to cancel from all daily activities”. (Emma)

While participants did not explicate on *why* they prioritized school or university over sport and exercise, we can assume that good grades and graduation might be connected to subsequent life and career options. In addition, research has also suggested that norms and expectations students have of themselves, such as being a good student, might also account for shifts in priorities [see also 30].

Another critical life event that accounted for the abandonment of sport activities due to new priorities was staying abroad either for leisure or study purposes. In those instances, it was more important to participants to travel and make new experiences. Again, Emma explained:

“Yes (..). So in Helsinki I didn’t do any sports. I had other things on my mind (laughs)”. (Emma)

Interestingly, most participants indicated that they did not pick up their previous sport activities upon their return, which might be due to subsequent transitions, such as starting to study or starting an apprenticeship and the associated re-allocations of scarce temporal resources.

Likewise, participants described moving to another city, which oftentimes also implied leaving the parental home, as a turning point when they shifted their priorities towards the experience of ‘arriving’ in a new place, living alone for the first time, and gaining independence and thus gave up their sport activities [see also 76]. Michael, for example, put it this way:

“Then just came the move and with it I would say a rapid decline [in sport behavior], because my weaker self knocked again and said: ‘Oh, okay, now so much is new. Hey, sport is not a priority for now. You don’t have to do so much sport.’”. (Michael)

This is also in line with Diehl and Hilger [77], who hypothesized that moving to a new city (e.g., when beginning to study) may require an initial orientation period in order to come to terms with new life circumstances. Especially those, who tried to keep up with sport activities in their hometown and had to commute between their new and old residence emphasized the issue of sorting out their priorities, which often led a subsequent drop out from sport. Ben, for instance, described how keeping the ties to his local sport club felt like a dull duty at some point as his new life as a student in a new city seemed to clash with his former sport engagement:

“Then, of course, things started to go downhill, because suddenly soccer was no longer just associated with the sport itself, but also with this permanent commuting. So, I would say that it already went down, it was more or less like: ‘Yes, okay, I’ll just do it now.’ Like a fulfillment of duty, actually, so I actually didn’t enjoy it like I used to.” (Ben)

When it came to becoming a parent, the few mothers and one father from our study indicated that they reduced or even abandoned their sport activities, which mirrors previous research findings [20, 22]. In this regard, Sandra pointed out how the use of her time came down to what she considered important:

“Ha, it feels like you never have time for sport when you have kids (laughs) (8s). Yes, but actually the time is already there, you just have to use it.” (Sandra)

Previous research has often considered this tendency under a time-use hypothesis, arguing that certain life events may influence discretionary time and thus lead to reductions in PA [12, 24, 70]. In contrast, we would suggest that, for example, increases in caring responsibilities lead to new priorities. This has also been hypothesized by Hull et al. [78], who argued that personal priorities and available temporal resources might be closely entangled as “parents may feel that attaining adequate physical activity no longer has the same importance, and with far less available time, physical activity may also become less feasible” (p. 6).

#### Finding purpose and becoming active (again)

Finally, while we have argued that critical life events and transitions often lead to a reduction in temporal resources (see Theme 1), participants also argued on multiple occasions that available time was less of an issue than setting priorities. For example, Lisa explained:

“I actually think that I would have always had time for it [sport activities] if I had wanted to. […] During my time in [city name], it was perhaps a bit lower. Actually, I think you always have time for it. That’s simply a matter of setting priorities (laughs)”. (Lisa)

In this regard, shifts in priorities might pull people away from sport activities even when they had time, but similarly, participants also described how some critical life events and transitions led them to experience a newly found motivation for these activities. Participants usually framed those events as stressful (in both a positive and negative sense) and as catalysts for discovering a meaning or purpose in sport, which subsequently made it a priority for them. In particular, they referred to giving birth and becoming a parent, starting to work or study, graduation, experiencing family disputes, experiencing illness, injuries, or ailments. In light of these events, several participants indicated that they associated sport with certain benefits, and, thus, purpose. Examples for identified purposes include taking some time for themselves or feeling more attractive (e.g., after giving birth), reducing stress and finding a balance (e.g., when starting to work or study), or reducing bodily complaints and becoming healthier (however, if injuries or illnesses were too severe, they rather led to abandonment of sport activities at least in the short-term). Ryan, who has two children, for instance, illustrated how he gained weight and needed some time for himself, which he found in sport:

“Because if you do nothing, then you also notice that somewhere grow a little, maybe around the hips or on the belly. So, I simply seized the initiative for myself and said: ‘I must do something again’. It also is a bit of a balance. Surely, with regard to everyday life, with stress, children, work. Exactly, so balance was also important for me, yes.” (Ryan)

This is in line with previous research considering that PA might act as a coping mechanism in light of stressful life events for men [23] and young women [24]. Our findings bring these considerations together, as they indicate that both men and women might use sport activities as a coping strategy, given they associate certain benefits with it and, thus, find a purpose in those activities and make them a priority. This has similarly been stated by Van Houten [30], who found that sport might offer an opportunity to deal with stress as life events occur.

In addition, while some events and transitions were associated with shifts in priorities motives and a decline in sport activities, participants, after some time, revalued sport and PA. Most notably, while participants associated becoming a parent with an initial decrease in daily PA and sport behavior, they–similar to the transition to secondary school–experienced a rebound effect after some time as they pursued more activities at lighter intensities, such as going for a walk [see also 74, 75]. Ryan, for example, pointed out that, as his children grew older, it was important to offer them something and to set a good and active example:

“Then with the children it has definitely (grins) increased, quite clearly. Because we are outside very often now. Recently, we even try to do more and see that we just go out with the children, look at the nature, see something like animals or we put the children on the back of the bike and so on, exactly. So, this is just increasing and we are also trying to be a bit more active ourselves.” (Ryan)

These findings are in line with research by Hamilton and White [26], who argued that parents generally experienced declines in PA, which were oftentimes exacerbated with the birth of a subsequent child, but who also stated that parenthood can be considered an opportunity to become (more) active, often, however, at lighter intensities. In addition, Colley et al. [28] reasoned that “the changing social roles associated with starting a family may often result in moves away from individualistic hobbies and outdoor pursuits to recreational activities as a couple or as a family” (p. 196), activities, which might help them cope with the demands of being a parent. Van Houten [30] similarly reports that new caring responsibilities and lighter daily PA might compensate or substitute sport activities.

### Theme 3: The (in)conveniences of context

In this theme, we elaborate on how the experience of critical life events and transitions might lead to changes in the physical environment, the available infrastructure (including sport offers), and social settings and relationships. In the first subtheme, *It’s not about whether people go*, *but where*, we discuss how the environmental and infrastructural context that is given to individuals with little to no room to change it may have a limiting or beneficial impact on daily PA and sport and exercise. In the second subtheme, *Sport and exercise are social behaviors*, we describe how social networks might evolve or disperse and thus lead to changes especially in sport and exercise behaviors.

#### It’s not about whether people go, but where

Experiencing critical life events and transitions, such as changing from one school to another, starting to study or work, or moving to a new city might lead to changes in physical environments and infrastructures that can be both favorable *or* unfavorable for daily PA. Participants described that, as distances became longer and it became more inconvenient to walk or take the bike to school, university, or work, it became more likely that they started travelling by public transport or car. For example, Leila stated:

“So, I just took the train and then the bus to school because it was an hour and a half away. Exactly, that was more of a cut [regarding daily PA] … Exactly, change of school, less cycling.” (Leila)

Participants also experienced declines in daily PA when the transition from one school to another was associated with a shorter distance, which decreased the amount of walking or cycling. In addition to distance, the topography appeared to be an important factor as participants indicated that they were less likely to walk or cycle if they had to go up a hill on their commuting route, as Sarah indicated:

“And here, I really only rode the bus. I lived on a mountain. Sometimes I rode my bike and it was really, really exhausting.” (Sarah)

In contrast, for others, the same critical life events and transitions could lead to increases in daily PA when infrastructure for active commuting improved. This was especially the case when life changes went hand in hand with an improvement in infrastructure for active commuting. This is in line with research, for example, on the transition to secondary school, which has often found an increase in active commuting [67, 79]. In addition, it is noteworthy that for most participants starting university required them to move to another city. Previous research has indicated this as a potentially relevant determinant for changes in PA-related behavior [70, 77]. While our results are in line with this assumption, they also emphasize that it depends on *where* and not only on *whether* people move. In this regard, participants explained that when the infrastructure was convenient, they covered even longer distances by foot or bike. These observations imply that even moving within one city might lead to substantial changes in daily PA if distance and topography or infrastructural circumstances change in an unfavorable way.

Additionally, with regard to sport activities, participants also experienced changes in physical environments and infrastructure as beneficial if they moved to another city or stayed abroad at a dorm and had facilities such as gyms directly available and oftentimes could work out there for free. Similarly, starting to study changed the availability of sporting offers as several participants described that the university sports offered them a wide variety of new and different sports with a low inhibition threshold to participate and at a reasonable price [see also 30]. John, for example, described his experience of studying abroad and reasoned that it was more convenient for him to go to the gym there, than it was back home:

“So just because it [the gym] was next to the dorm (laughs). That was the reason. Didn’t cost anything and it was next to the dorm. Because that’s great in [country]. Because they have the whole university sports stuff more or less all included in the fees somehow and they’re spread all over the city. There are somehow five fitness studios belonging to the university and swimming pools”. (John)

#### Sport and exercise are social behaviors

Most of our participants engaged in some sort of club-based sport during childhood and adolescence, which is the most common social setting for sport activities in Germany. As they grew older, they participated more informally in sport and exercise with friends. Participants regularly described two unfolding dynamics in these two settings, which led to a subsequent drop out from these activities. First, they explained that their sport endeavors ended as coaches, teammates, or training partners dropped out due to critical life events and transitions in their own lives, such as moving away, starting an apprenticeship or to study, or sustaining an injury. Participants often described that losing a reference person and friend who held them accountable to go to training, led to a subsequent loss in motivation, as they did not want to train alone. In this regard, Viola described:

“I’m there with a friend, because we thought we’d try it. And then it was actually quite fun. And, yes, then we did it again for a semester. But then she left the university and then I didn’t want to do it alone anymore (laughs).” (Viola)

This relates to research, which has stated that adolescents’ exercise behavior is related to exercise behavior of their peers and has thus emphasized the relevance of social networks for exercise maintenance [80]. For adults, findings regarding social support from friends and peers seem to be more inconclusive [81], but findings from a group exercise intervention study suggest that “being part of a group with similar abilities brought a sense of comfort to all participants and appeared to facilitate feelings of competence and the confidence of adopting and continuing with the program” [82, p. 14]. Second, participants sometimes experienced new coaches as a challenge. This was even more pronounced when a new coach confronted them with a different set of expectations. Participants indicated on several occasions that they had to get used to a new coaches’ demands, style, and training routines, which often led to a dropout from sports. This connects to previous research that has shown that the quality of coach-athlete relationships is crucial for a continued participation in sport activities [e.g., 83, 84].

In contrast, participants also indicated that beginning to study and meeting new friends at university favored engaging in some sporting activity together. This relates to research stating that friends might provide crucial social support for PA in university and college students [85]. Brad, for example, explained:

“Then I met a friend at university and then we just went running together, we did yoga together, we went bouldering together, something like that.” (Brad)

### Strengths, limitations, and future research

This is, to the best of our knowledge, one of the first qualitative studies to assess the role of *critical* (i.e., subjectively relevant) life events and transitions with regard to *two PA domains*, namely daily PA and sport and exercise behavior. While especially quantitative studies have indicated that certain life events and transitions are associated with changes in PA and sport and exercise behavior, this strand of research was not able to unveil the underlying dynamics of these behavioral adaptations. Our theoretical and methodological approach offers an in-depth analysis of these behavioral and attitudinal changes in several regards. First, our qualitative approach allows identifying underlying dynamics and not only describe in which ways PA behaviors might change. Second, the focus on *critical* life events and transitions allows putting a lens on people’s experiences and what they considered to be important instead of using pre-selected life event scales or sampling only participants who find themselves in a given transition. Interestingly, some life events that research has previously deemed important (e.g., entering a relationship, ending a relationship, starting cohabitation, getting married, or ending a job) were not mentioned by our participants. This might be due to a lack of experience of these events or the importance of these events for PA behavior has been overstated in previous studies. In addition, our qualitative focus on subjectively relevant life events and transitions yielded the insight that it is not only the ‘big’ life events, but also smaller events and events experienced by others that are valued as significant and PA altering. Third, our study design allows to assess the entire life span, while integrating an interview approach with a drawing activity. The latter encouraged participants to describe trajectories and developmental patterns in relation to life events they considered important. This often elicited further explanations and helped to better understand when, why, and how behaviors and associated attitudes changed. In addition, this approach allowed us to assess life events and transitions with regard to their temporal structure, as long-term developments might otherwise remain concealed leading to the false conclusion that behavioral changes in the context of critical life events and transition might be permanent.

Limitations include that our study sample consisted of mainly young, well-educated, and healthy adults, which limits our findings to this particular group. In this regard, participants had for example rarely experienced severe illnesses or impairments. Future research might study different participant groups with similar methods. A second limitation might be that the method we applied might lead participants to think more on an associative level. This means that participants simply associate certain life events and transitions with more or less daily PA or sport and exercise without offering any explanations, or they explained that other factors instead of the associated event or transition actually accounted for behavioral adaptations (e.g., daily PA might drop during high school, but it is actually a change in leisure interests that leads to the decline). However, conducting our reflexive thematic analysis in a rigorous and coherent way, we aimed to counteract jumping to superficial conclusions. In addition, participants sometimes only *named* life events that accounted for changes in daily PA, sport, and exercise instead of elaborating on them (e.g., pregnancy or injury and illness). In these instances, we were not able to identify any underlying mechanisms across the data set, which does however not mean that they do not exist. Future research might, in this regard, further elaborate on the associated dynamics of these events and transitions.

## Conclusion

Previous research has indicated that similar life events can affect different PA domains and that there is large inter-individual variation in the ways individuals adapt their behavior [10, 23]. This study is, to the best of our knowledge, the first that explicitly examines the *underlying dynamics* of life events and transitions that lead to changes in different PA domains (i.e. daily PA and sport and exercise behavior) from a subjective perspective. Our findings both reflect previous research and offer new insights as to when, why, and how critical life events and transitions are associated with (re-)adjustments in PA-related behavioral patterns and attitudes.

The three developed themes (1) *The finitude of temporal resources*; (2) *The plasticity of priorities and motives*; and (3) *The (in)conveniences of context* reflect the ambivalent relationship between critical life events and transitions and daily PA and sport and exercise. On the one hand, they reaffirm previous hypotheses that reallocations of temporal resources, shifts in priorities [20–22], or alterations in contextual circumstances [16–19] may lead to adaptations in PA-related behavior. On the other hand, the themes highlight that critical life events and transitions cannot universally be labelled as barriers to or promoters of daily PA and sport and exercise. Rather, whether a life event or transition accounts for the uptake or abandonment of or increases or decreases in those activities depends on the extent to which events are considered subjectively significant (meaning whether they are *critical*) and which kind of life changes (i.e., dynamics) they trigger.

In general, the three underlying dynamics that we present here are closely inter-related, may influence each other, and can even occur at the same time or shortly after each other. For example, shifts in institutionalized requirements may result in new obligations and demands, which limit temporal resources or increase sedentary time. However, the degree to which a lack in temporal resources or an increase in sedentary behavior has an impact on PA-related behaviors depends on institutional contexts and their properties as well as when and at what age the transition into these contexts occurs in the life span. In addition, we would argue that the use of leisure time represents an issue of personal priorities and motives, which can similarly be affected by critical life events and transitions. In this sense, the willingness to invest time in daily PA or sport and exercise depends on how subjectively relevant other life domains are currently (e.g., upcoming graduation, examination periods, etc.). Finally, even if individuals were (not) motivated to be physically active, environmental and infrastructural circumstances as well as social settings and relationships might change due to critical life events and transitions and hinder (or assist) them in their endeavors. Eventually, the extent of behavioral adaptations depends on the degree to which a given equilibrium between institutionalized demands and obligations, individual priorities and motives, and contextual factors is disturbed or shattered. This is in line research, which has argued that for emerging adults, life events lead to new life situations, a more adult status, and, consequently, a conflict in temporal, social, physical, mental, and economic resources that might lead to constraints, but also new opportunities as resources and priorities become re-evaluated and re-balanced [30].

Additionally, our results suggest that critical life events and transitions may not only lead to the uptake or increase and abandonment or decrease of PA-related behaviors, but also to the substitution of one PA domain with another. In addition, various critical life events and transitions may occur at the same time, simultaneously to other concurrent developments (e.g., puberty), or in short succession and at different subjective ‘intensities’. However, whether and to which extent this leads to the described underlying dynamics and thus changes in daily PA or sport and exercise might depend on the timing and the interdependencies between related life domains and how they compete for resources [22].

Like previous research, our results show that critical life events and transitions are natural interventions that can occur anytime across the life span [10, 37] and that young adulthood is a developmental period where various life events and transitions are likely to co-occur or occur in short succession [8–10]. In addition, our findings emphasize that critical life events and transitions are socially embedded phenomena [37] and that those contextual factors are relevant as to when, why, and how people change their PA-related behaviors [29]. Children, adolescents, and young adults might be more likely to decrease or abandon PA if it does not fit into their overall life concept or match their motives. For instance, if individuals value sport and exercise as important activities and attribute meaning to them, they appear to be more willing to invest time despite potential temporal constraints. On the other hand, if other life domains, such as family, work, or education, are more important, they will be less likely to keep up their engagement.

Eventually, there is not a straight causal link between life events and behavior. Our results emphasize that critical life events and transitions can have a tremendous impact on temporal resources, individual priorities and motives, and contextual circumstances, such as the physical environment and social settings and relationships. Consequently, changes in these areas can lead to individual adaptations in daily PA or sport and exercise behavior and related attitudes.

## Supporting information

S1 Table Overview of all assessed developmental trajectories for the biographical mapping. (DOCX)
