# Supplementary material for: Ways into physical (in)activity: The role of critical life events and transitions in the reconstructions of young adults
Source: PLoS One. 2023 Aug 22;18(8):e0290438. doi: 10.1371/journal.pone.0290438 (PMC10443846; doi:10.1371/journal.pone.0290438)
Supplement: S1 Table — (DOCX) [file pone.0290438.s001.docx]

Supplementary Material

**S1 Table.** Overview of all assessed developmental trajectories for the biographical mapping

| Health- and activity-related developmental trajectories |
| --- |
| Amount of PA in everyday life |
| Amount of sport and exercise |
| Relevance of sport and exercise |
| Enjoyment of sport and exercise |
| Health behavior |
| Physical fitness in everyday life (i.e. general fitness level) |
| Exercise-related physical fitness (i.e. athletic fitness level or training status) |
| Physical complaints |
| Psychological stress |
| Global well-being |
| Attractiveness |
| Social embeddedness |
| Relevance of sport and exercise in one’s own social environment |
| Organization of everyday life |
| Temporal resources for sport and exercise |
